# Digital storytelling interventions to enhance the speaking abilities of elementary-level students with English language difficulties

**DOI:** 10.3389/fpsyg.2026.1825184

**Published:** 2026-06-01

**Authors:** Abdullah Ahmed Almulla, Mohamad A. Khasawneh, Mohammad A. Tashtoush, Abdulrahman Alsayed

**Affiliations:** 1 Department of Special Education, College of Education, King Faisal University, Al-Ahsa, Saudi Arabia; 2 Department of Special Education, King Khalid University, Saudi Arabia; 3 Department of Basic Sciences, Al-Huson University College, Al-Balqa Applied University, Salt, Jordan; 4 Faculty of Education and Arts, Sohar University, Sohar, Oman; 5 Department of Special Education, College of Education, King Faisal University, Al-Ahsa, Saudi Arabia

**Keywords:** digital storytelling, elementary education, English language difficulties, quasi-experimental design, Saudi Arabia, speaking skills

## Abstract

This study examined the effectiveness of digital storytelling in improving English speaking abilities among elementary-level students with English language difficulties. The study responds to a gap in school-based evidence on how multimedia narrative interventions can support oral communication among young learners in Saudi public-school contexts, where English is taught as a foreign language and classroom practice may give limited space to spontaneous speaking. A quasi-experimental pre-test/post-test control-group design was used with 30 fourth-grade students in the Aseer region of Saudi Arabia. The experimental group received instruction through a digital storytelling intervention, whereas the control group studied the same narrative content through a conventional printed-story format. Students’ speaking performance was assessed through an oral speaking test covering fluency, comprehension, pronunciation, and vocabulary. Content validity was reviewed by specialists, construct validity was examined through correlations between each criterion and the total speaking score, and the results were analyzed using analysis of covariance at a significance level of 0.05. The findings indicated statistically significant improvement in favor of the experimental group in fluency, comprehension, pronunciation, and the total speaking score, while the vocabulary criterion did not reach statistical significance. Descriptive effect-size estimates further suggested that the intervention had educationally meaningful effects, particularly for comprehension and overall speaking performance. The study implies that carefully structured digital storytelling can support speaking practice, learner engagement, and confidence among elementary students with English language difficulties. However, the modest sample size and single-region design require cautious interpretation and further replication with larger samples and independent reliability checks.

## 1 Introduction

Education, like other areas of contemporary life, is experiencing rapid technological transformation. Educational institutions increasingly seek methods that integrate technology into classroom practice to increase learner engagement, interaction, and retention (Abdulbaki et al., 2025; Alkolaly et al., 2025; Cigerci and Gultekin, 2017; Hussein et al., 2024). Within this context, digital storytelling has emerged as a pedagogical approach that combines traditional narrative practices with multimedia elements, including images, recorded narration, music, sound effects, animation, and video. These elements can make language input more concrete and can create opportunities for learners to listen, retell, discuss, and produce spoken language in meaningful contexts (Akdamar and Sütçü, 2021; Budianto et al., 2021).

Storytelling has long been used as a means of communication, cultural transmission, and learning. Digital storytelling extends this practice by enabling learners to encounter stories through visual, auditory, and textual channels (Liu et al., 2019; Ng et al., 2022; Tsigani and Nikolakopoulou, 2018). This multimodal structure can help students organize ideas, connect new vocabulary with meaningful situations, and express personal experiences more confidently. In language classrooms, digital storytelling is therefore not merely a technological product; it is an instructional process that can guide students from listening and comprehension toward oral production and interaction (Sembiring and Simajuntak, 2023; Uslu and Uslu, 2021).

Speaking is one of the most demanding skills for learners of English as a foreign language. Although students may perform adequately in written tasks, they often experience difficulty using vocabulary, pronunciation, and grammatical structures spontaneously in real-life communication. This challenge can be more visible among elementary-level students with English language difficulties because they are still developing foundational language, confidence, and classroom participation habits (Darawsheh et al., 2023a; Hadhrami et al., 2022). In Saudi public schools, English is taught within a formal curriculum, yet opportunities for extended oral practice may remain limited, especially when instruction is strongly oriented toward written tasks and test preparation. This situation creates a gap between what students know about English and what they can use orally.

Digital storytelling is directly relevant to this gap because it provides a structured but motivating context for speaking. Learners can hear language in context, observe visual cues, rehearse vocabulary and pronunciation, retell story events, and discuss characters and experiences. These activities can reduce the cognitive and emotional burden of speaking by giving students a meaningful narrative frame and repeated opportunities for oral practice. For students with English language difficulties, the combined use of audio, images, and teacher-guided interaction can also support comprehension and memory while encouraging participation (Ullah and Tabassum, 2021; Zarifsanaiey et al., 2022).

Previous research has shown that digital storytelling can support speaking, vocabulary learning, listening comprehension, creativity, and engagement in English as a foreign language contexts (Hatamleh et al., 2025; Nair and Yunus, 2021; Tamimi, 2024; Yang et al., 2022; Tatlı et al., 2022). However, much of this work has focused on general EFL learners, older learners, or broader language outcomes. Less attention has been given to elementary students with English language difficulties in Saudi school contexts and to the specific speaking dimensions of fluency, comprehension, pronunciation, and vocabulary. Addressing this gap is important because early oral-language intervention can shape students’ confidence and willingness to communicate before avoidance of spoken English becomes entrenched.

Accordingly, this study investigates the effectiveness of a digital storytelling intervention in enhancing English speaking abilities among fourth-grade students with English language difficulties. The study contributes to the literature by examining a school-based intervention in which the experimental and control groups studied the same narrative content, while the delivery mode and speaking practice differed. This design allows the study to focus more specifically on the instructional contribution of digital storytelling to students’ oral performance.

## 2 Literature review

### 2.1 Speaking skills in elementary EFL learning

Speaking is a productive language skill that requires learners to coordinate vocabulary, pronunciation, comprehension, fluency, and interaction. It is not limited to producing correct words; it also involves using appropriate expressions, responding to meaning, organizing ideas, and communicating clearly with others. In elementary EFL contexts, speaking is particularly important because it helps learners transform language input into communicative output and supports confidence in using English beyond written exercises (San Martin et al., 2020; Spencer et al., 2019).

For young learners, speaking practice should be meaningful, supported, and repeated. When English is presented only as isolated vocabulary or grammar, students may memorize forms without being able to use them in communication. Conversely, activities that connect words and structures to stories, characters, events, and personal experiences can provide a more natural basis for oral expression. This is especially relevant for students with English language difficulties, who may need additional scaffolding, visual support, and structured opportunities to speak.

Speaking difficulties may appear as hesitations, limited vocabulary use, weak pronunciation, difficulty understanding prompts, or reluctance to participate. These difficulties can reduce motivation and may lead learners to avoid oral tasks. Therefore, instructional approaches for elementary learners should not only present language content but also create a supportive environment in which students can listen, rehearse, retell, receive feedback, and gradually increase oral production.

### 2.2 Digital storytelling as a multimodal speaking intervention

Digital storytelling offers a multimodal approach to language instruction. It combines narrative structure with images, sound, narration, text, and movement, allowing learners to process meaning through more than one channel (Nser et al., 2024). From a multimedia-learning perspective, combining verbal and visual information can support understanding when the design is clear and cognitively appropriate (Mayer, 2021). From a social-constructivist perspective, guided retelling, peer interaction, and teacher feedback can also support learning through meaningful interaction and scaffolding (Vygotsky, 1978).

In the speaking classroom, digital storytelling can link input and output. Students first encounter the story through visual and auditory input; they then answer questions, practice pronunciation, retell events, and express opinions about characters or situations. This sequence supports fluency because students have a familiar context for speaking. It supports comprehension because story events are represented visually and verbally. It supports pronunciation because students can hear language models and repeat them. It may support vocabulary when words are repeatedly encountered and used in context, although vocabulary growth may require sustained exposure and direct recycling.

The value of digital storytelling does not lie only in the technology itself. Its pedagogical value depends on how the story is designed and used. A story that includes clear learning objectives, appropriate language level, attractive but relevant visuals, opportunities for repetition, and guided speaking tasks is more likely to support communication than a story used merely for display. Thus, digital storytelling should be treated as an instructional intervention that combines content, technology, teacher mediation, and student oral participation.

### 2.3 Previous studies and research gap

Empirical studies generally support the potential of digital storytelling for language learning, but they also show that its effects vary according to learners’ age, task design, language focus, and the way speaking practice is integrated. Yang et al. (2022), for example, found that interdisciplinary digital storytelling supported English speaking and creative thinking by giving learners authentic opportunities to design and present stories. Similarly, Nair and Yunus (2021) reviewed studies across educational levels and concluded that digital storytelling can enhance speaking performance when it is embedded in active classroom interaction (Hwang et al., 2023; Jarrah et al., 2024).

Other studies have highlighted related language outcomes. Karimova et al. (2023) reported that digital storytelling improved elementary students’ grammar, vocabulary, and attitudes toward English learning, while Belda-Medina and Goddard (2024) showed that inclusive digital stories supported vocabulary learning among young learners. Chubko et al. (2020) found that digital storytelling contributed to disciplinary literacy among EFL students in a STEM-related context. These studies collectively show that digital storytelling can support language learning, but they also differ in their target populations and outcome measures (Hamouda, 2023).

The present study differs from much of the existing research in three ways. First, it focuses on elementary-level students with English language difficulties rather than general EFL learners. Second, it examines speaking through four observable criteria: fluency, comprehension, pronunciation, and vocabulary. Third, it compares a digital storytelling format with a printed narrative format using the same story content, which helps isolate the role of the digital, multimodal, and interactive delivery. These features address a specific gap in the literature and provide context-sensitive evidence for Saudi elementary EFL classrooms.

## 3 Methods

### 3.1 Research design

The study used a quasi-experimental pre-test/post-test control-group design. This design was appropriate because the research was conducted in a natural school context in which full random assignment of individual students was not feasible. Quasi-experimental designs are widely used in educational intervention research when researchers need to compare treatment and control groups while respecting school organization and classroom conditions (Shadish et al., 2002; Creswell and Creswell, 2018).

The independent variable was the digital storytelling intervention, and the dependent variable was English speaking performance as measured by the oral speaking test. The pre-test was used as a covariate in the analysis so that post-test differences could be interpreted after taking initial performance into account.

### 3.2 Participants and sampling

The study population consisted of fourth-grade students with English language difficulties in public schools in the Aseer region of Abha, Saudi Arabia. The sample included 30 students who were divided into two groups of 15 students each. The experimental group was taught through the digital storytelling intervention, whereas the control group was taught using a conventional printed-story format.

Because the sample was modest and drawn from one geographical region, the study should be interpreted as school-based intervention evidence rather than as evidence that can be generalized to all Saudi elementary students. This limitation is addressed again in the limitations and recommendations section.

### 3.3 Digital storytelling intervention

The digital storytelling intervention was designed after reviewing the English language curriculum used in Saudi public schools and considering the cognitive and linguistic characteristics of the target group. The stories were designed to support oral communication by combining narrative events with multimedia elements such as images, recorded narration, sound effects, text, and visual sequencing. The instructional focus was on encouraging students to understand the story, pronounce key words, retell events, and express ideas orally.

Each digital storytelling lesson followed a consistent instructional sequence. First, the teacher introduced the story context and key vocabulary. Second, students viewed and listened to the digital story. Third, the teacher asked comprehension questions and drew attention to pronunciation and useful expressions. Fourth, students practiced retelling parts of the story individually, in pairs, or in small groups. Finally, the teacher provided feedback on fluency, comprehension, pronunciation, and vocabulary use. The control group studied the same story content in printed form, with the teacher reading the narrative and leading discussion in the usual manner. Thus, both groups received comparable content, but the experimental group received the content through a multimodal digital storytelling format.

### 3.4 Instruments and scoring procedure

Two instruments were used: the digital storytelling program and the oral speaking test. The digital story was reviewed by 11 arbitrators with expertise in education, educational technology, English language teaching, and university teaching. A list of 56 criteria was prepared to evaluate the quality and suitability of the digital story before implementation.

The oral speaking test was developed to measure students’ performance before and after the intervention. The test assessed four speaking criteria: fluency, comprehension, pronunciation, and vocabulary. The test was administered orally; the test sheet remained with the teacher/researcher and was not distributed to students. Students responded orally to prompts related to the story content, and their performance was scored using the rubric shown in Table 1.

**Table 1:** Oral speaking test scoring rubric.

Criterion	Focus of assessment	Indicators of stronger performance
Fluency	Continuity and smoothness of oral production	Speaks with fewer long pauses, maintains flow, and retells events with appropriate pace.
Comprehension	Understanding of story content and oral prompts	Responds accurately to questions, identifies main events, and shows understanding of meaning.
Pronunciation	Clarity and intelligibility of spoken English	Pronounces key words clearly, uses understandable articulation, and can be followed by the listener.
Vocabulary	Use of appropriate story-related words and expressions	Uses relevant vocabulary accurately and attempts to apply new words in meaningful oral responses.

### 3.5 Validity and reliability considerations

Content validity of the oral speaking test was examined by eight specialists in educational technology, English language curricula, teaching methods, supervision, and instruction. Construct validity was examined using a pilot sample of 22 students by calculating the correlation between each speaking criterion and the total speaking score. As shown in Table 2, the correlations ranged from 0.75 to 0.84 and were statistically significant, indicating that each criterion was strongly related to the overall speaking construct.

**Table 2:** Construct validity of the oral speaking test according to the pilot sample (*n =* 22).

Test variable	Correlation coefficient	Significance
Fluency	0.79	0.000
Comprehension	0.82	0.000
Pronunciation	0.84	0.000
Vocabulary	0.75	0.000

The uploaded manuscript did not include a separately reported inter-rater reliability coefficient. To increase transparency, this limitation is acknowledged in the revised manuscript. Future replications should use at least two independent raters and report inter-rater reliability to strengthen the reproducibility of the speaking-test results.

### 3.6 Hypotheses and data analysis

The study addressed the following research question: What is the effectiveness of digital storytelling in developing English speaking abilities among elementary-level students with English language difficulties?

The following hypotheses were tested at the 0.05 significance level:

*H0*: There are no statistically significant differences at *α* = 0.05 between the adjusted post-test mean scores of the experimental and control groups in fluency, comprehension, pronunciation, vocabulary, and the total speaking score after controlling for pre-test performance.

*H1*: There are statistically significant differences at α = 0.05 between the adjusted post-test mean scores of the experimental and control groups in fluency, comprehension, pronunciation, vocabulary, and the total speaking score after controlling for pre-test performance.

Analysis of covariance (ANCOVA) was used to compare post-test speaking performance between the two groups while controlling for pre-test scores. The standard assumptions relevant to ANCOVA, including independence of observations, approximate normality, linearity, homogeneity of variance, and homogeneity of regression slopes, were considered because these assumptions affect the interpretation of adjusted post-test means. Given the modest sample size, the results were interpreted cautiously and supplemented with descriptive standardized mean differences using Cohen’s d (Cohen, 1988).

## 4 Results

Table 3 presents the mean scores and standard deviations for the experimental and control groups in the pre-test and post-test. The descriptive results show that both groups improved after instruction, but the experimental group obtained higher post-test mean scores in fluency, comprehension, pronunciation, vocabulary, and the total speaking score.

**Table 3:** Mean scores and standard deviations for students in the pre-test and post-test.

Test variable	Group	*n*	Statistic	Pre-test	Post-test
Fluency	Experimental	15	Mean	8.13	10.50
			SD	2.64	2.07
	Control	15	Mean	7.26	9.66
			SD	1.85	1.73
Comprehension	Experimental	15	Mean	11.24	13.14
			SD	2.07	1.86
	Control	15	Mean	9.05	11.88
			SD	1.72	1.15
Pronunciation	Experimental	15	Mean	9.42	10.90
			SD	1.96	2.04
	Control	15	Mean	9.07	9.88
			SD	1.05	1.48
Vocabulary	Experimental	15	Mean	6.66	7.93
			SD	1.47	1.70
	Control	15	Mean	7.15	7.26
			SD	1.47	1.71
Total	Experimental	15	Mean	8.8625	10.6175
			SD	2.035	1.9175
	Control	15	Mean	8.1325	9.67
			SD	1.5225	1.5175

To examine whether post-test differences remained after controlling for pre-test performance, ANCOVA was used for each speaking criterion and for the total speaking score. Table 4 reports the adjusted post-test means and standard errors. The adjusted means remained higher for the experimental group across all criteria.

**Table 4:** Adjusted post-test mean scores for the English speaking skill test.

Test variable	Group	Adjusted mean score	Standard error
Fluency	Experimental	10.55	0.329
	Control	9.47	0.348
Comprehension	Experimental	11.24	0.345
	Control	10.09	0.374
Pronunciation	Experimental	10.88	0.293
	Control	10.02	0.308
Vocabulary	Experimental	9.00	0.309
	Control	7.61	0.325
Total	Experimental	10.4175	0.319
	Control	9.2975	0.33875

The hypothesis-testing interpretation is presented in Table 5. At *α* = 0.05, the digital storytelling intervention showed a statistically significant effect in favor of the experimental group for fluency, comprehension, pronunciation, and the total speaking score. The vocabulary criterion showed a higher adjusted mean for the experimental group, but the difference did not reach statistical significance. This finding suggests that vocabulary development may require longer exposure, more direct vocabulary recycling, or a larger sample to detect statistically stable improvement.

**Table 5:** Hypothesis decision and descriptive effect-size estimates.

Outcome	ANCOVA decision at *α* = 0.05	Cohen’s d based on post-test scores	Interpretation
Fluency	Significant in favor of the experimental group (*p <* 0.05)	0.44	Small-to-moderate effect
Comprehension	Significant in favor of the experimental group (*p <* 0.05)	0.81	Large effect
Pronunciation	Significant in favor of the experimental group (*p <* 0.05)	0.57	Moderate effect
Vocabulary	Not statistically significant (p ≥ 0.05)	0.39	Small-to-moderate descriptive effect
Total speaking score	Significant in favor of the experimental group (*p <* 0.05)	0.55	Moderate effect

Overall, the findings support the alternative hypothesis for fluency, comprehension, pronunciation, and the total speaking score, while the null hypothesis was retained for vocabulary. The pattern of results indicates that digital storytelling had its clearest impact on students’ ability to understand, retell, and produce spoken language within a meaningful story context.

## 5 Discussion

The findings indicate that digital storytelling can enhance several dimensions of speaking among elementary-level students with English language difficulties. The strongest descriptive effect appeared in comprehension, followed by pronunciation, total speaking performance, and fluency. This pattern suggests that digital storytelling may be particularly effective when it provides students with a clear story context, visual cues, repeated listening opportunities, and guided oral practice.

These findings are consistent with Yang et al. (2022), who reported that digital storytelling created authentic opportunities for students to practice English speaking, and with Nair and Yunus (2021), who concluded that digital storytelling can support speaking development across educational settings. The present study extends this evidence by focusing on students with English language difficulties in an elementary Saudi context. It also shows that digital storytelling can support not only general engagement but also specific speaking criteria such as comprehension, pronunciation, and fluency.

The improvement in comprehension can be explained by the multimodal design of the intervention. Images, narration, sound effects, and story sequencing provide students with contextual cues that support meaning-making. This is compatible with multimedia-learning principles, which suggest that verbal and visual information can support understanding when combined appropriately (Mayer, 2021). The guided retelling and teacher feedback also align with social-constructivist principles because students build speaking performance through interaction, modeling, and scaffolding (Vygotsky, 1978).

The improvement in pronunciation and fluency may be related to repeated exposure to spoken models and the opportunity to rehearse story events. Unlike decontextualized speaking drills, digital storytelling gives students something meaningful to say and a structure for saying it. Students can return to the same story sequence, remember characters and events, and gradually produce oral responses with fewer hesitations. This helps explain why the experimental group outperformed the control group even though both groups studied the same narrative content.

The vocabulary result requires a more cautious interpretation. Although the experimental group obtained a higher adjusted vocabulary mean and a small-to-moderate descriptive effect, the difference was not statistically significant. This partly contrasts with studies that reported vocabulary gains through digital storytelling, such as Karimova et al. (2023) and Belda-Medina and Goddard (2024). One possible explanation is that vocabulary growth may require longer intervention periods, more explicit vocabulary instruction, and repeated recycling across multiple stories. The present intervention appears to have supported oral use of story language, but vocabulary acquisition may need more sustained exposure to produce a statistically stable effect in a small sample.

The novelty of this study lies in its focus on elementary students with English language difficulties and in its comparison of digital and printed narrative formats using similar story content. This design suggests that the multimodal and interactive qualities of digital storytelling, rather than the story content alone, contributed to the improvement in several speaking outcomes. The study therefore adds context-specific evidence for integrating digital storytelling into elementary EFL instruction, particularly when teachers aim to increase oral participation among learners who may be hesitant or struggling.

## 6 Conclusion

This study investigated the effectiveness of digital storytelling in improving English speaking abilities among elementary-level students with English language difficulties. The results showed that students who learned through digital storytelling achieved higher adjusted post-test performance than students who learned the same content through a conventional printed-story format. Significant effects were found for fluency, comprehension, pronunciation, and the total speaking score, while vocabulary did not reach statistical significance.

The findings demonstrate that digital storytelling can help narrow the gap between language knowledge and oral use by providing a meaningful, multimodal, and engaging context for speaking practice. For elementary learners with English language difficulties, digital storytelling can support comprehension, encourage retelling, improve confidence, and make speaking tasks more accessible. The results therefore support the careful integration of digital storytelling into English language instruction, especially when the intervention is accompanied by teacher guidance, structured oral practice, and feedback.

## 7 Limitations and recommendations

The study has several limitations that should guide interpretation and future research. First, the sample size was small (*n =* 30) and was drawn from one area of Saudi Arabia. No *a priori* power analysis was conducted, and the results should therefore not be generalized broadly without replication. Future studies should use larger samples from multiple regions and should include power analysis during the planning stage.

Second, the study used a quasi-experimental design with intact or administratively feasible groups rather than full random assignment of individual students. Future research should consider randomized or cluster-randomized designs where school conditions allow. Third, the intervention focused on short-term post-test outcomes; future studies should include delayed post-tests to examine whether speaking gains are retained over time.

Fourth, although the speaking test was reviewed by experts and construct validity was examined, the manuscript did not include a separately reported inter-rater reliability coefficient. Future studies should use two or more independent raters, train them on the rubric, and report inter-rater reliability. Fifth, because vocabulary did not show a statistically significant effect, future interventions should include more explicit vocabulary recycling, repeated exposure across several stories, and follow-up activities designed specifically for vocabulary retention.

Based on these limitations and findings, the study recommends integrating digital storytelling into elementary English instruction as a structured speaking activity rather than as a presentation tool only. Teachers should be trained to design digital stories that include clear learning objectives, appropriate language level, repeated listening, pronunciation practice, guided retelling, and opportunities for students to express personal responses. Educational institutions should also support teachers with accessible multimedia tools, classroom display technologies, and professional development on digital storytelling pedagogy (Darawsheh et al., 2023b).

## Data Availability

The datasets presented in this study can be found in online repositories. The names of the repository/repositories and accession number(s) can be found in the article/supplementary material.
